# Antidiabetic effects of *Brugmansia aurea* leaf extract by modulating the glucose levels, insulin resistance, and oxidative stress mechanism

**DOI:** 10.3389/fnut.2022.1005341

**Published:** 2022-10-11

**Authors:** Nisar Fatima, Fareeha Anwar, Uzma Saleem, Aslam Khan, Bashir Ahmad, Irum Shahzadi, Hammad Ahmad, Tariq Ismail

**Affiliations:** ^1^Faculty of Pharmacy, Riphah Institute of Pharmaceutical Sciences, Riphah International University, Lahore, Pakistan; ^2^Department of Pharmacology, Faculty of Pharmaceutical Sciences, Government College University, Faisalabad, Pakistan; ^3^Hamza College of Pharmaceutical and Allied Health Sciences, Lahore, Pakistan; ^4^Department of Biotechnology, Comsat University Islamabad Abbottabad Campus, Abbottabad, Pakistan; ^5^Bashir Institute of Pharmaceutical Sciences, Islamabad, Pakistan; ^6^Department of Pharmacy, Comsat University Islamabad Abbottabad Campus, Abbottabad, Pakistan

**Keywords:** alloxan, oxidative stress, insulin resistance, HbA1c, glucose

## Abstract

**Background:**

*Ethnopharmacological relevance: Brugmansia*, a genus of the Solanaceae family, has historically been utilized in many different parts of the world as an anti-inflammatory for treating skin infections, wounds, and bodily aches and pains. The current study aimed to investigate the potential benefits of a methanolic extract of *Brugmansia aurea* in the management of diabetes and underlying complications in alloxanized-induced diabetic rats.

**Materials and methods:**

Animals were divided into nine groups (*n* = 6). Four groups received different standard oral hypoglycemic agents; three groups received 100, 200, and 400 mg/kg of *B. aurea* leaf extract for six consecutive weeks, and the remaining two were normal and disease control groups. All groups received alloxan (150 mg/kg) except for the normal control. Only those animals whose glucose levels were raised to 200 mg/dl were selected for the study. After a 6-week dosage period, various biochemical parameters, as well as HbA1c, antioxidant profile, oral glucose tolerance test (OGTT), insulin sensitivity, histopathology, and insulin resistance, were measured and compared with the untreated diabetic group.

**Results:**

*Brugmansia aurea* leaf extract at a dose of 400 mg/kg showed potent antidiabetic activity by reducing blood glucose levels (*p* < 0.001) after 6 weeks of treatment. OGTT data showed that *B. aurea* exhibited significant (*p* < 0.001) glucose tolerance by significantly reducing blood glucose levels in just 2 h post-treatment. Other tests showed that plant extract significantly increased (*p* < 0.001) insulin sensitivity and decreased (*p* < 0.001) insulin resistance. The biochemical profile showed reduced triglyceride and cholesterol, while the antioxidant profile showed restoration of antioxidant enzymes in the pancreas, kidney, and liver tissues of treated rats.

**Conclusion:**

The present study indicated that crude extracts of *B. aurea* increase insulin sensitivity and reduce hyperlipidemia in diabetic rats, which rationalizes the traditional medicinal use of this plant as an antidiabetic agent.

## Introduction

The term “diabetes mellitus” was first used and documented by the Egyptians and Greeks; this term means to excrete sweetness from the body. Diabetes deprives the body of its capacity to manage blood glucose concentrations, resulting in long-term pathological problems such as cardiovascular abnormalities, nephropathy, and neuropathy, among others (1). Blood glucose regulation works as a negative feedback mechanism based on pancreatic hormones, i.e., insulin and glucagon. High blood glucose concentrations in the body trigger pancreatic β-cells to release insulin, which signals the liver to store excessive glucose in glycogen form. In the body, adipose cells or skeletal muscles take up glucose by translocating GLUT4 receptors that are present on the cell surface. Decreased the blood glucose concentration triggers the α-subunit of insulin receptors to produce glucagon, signaling the liver to convert stored glycogen into glucose to return to the normal physiological state (2).

Insulin resistance (IR) is the major complication associated with diabetes (3) and is characterized by the impaired response to several biological mechanisms, i.e., downregulation of the receptor, abnormal GLUT-4 function, defects in ser/thr residues of insulin receptors genetically, defects in insulin receptor proteins, and abnormal functioning of PIP3 (4). The defects in the insulin signaling pathway may occur in different forms, i.e., some forms of protein kinase C (PKC) modulate the insulin signaling pathway and cause free fatty acid-induced insulin resistance. Similarly, excessive phosphorylation of serine residues decreases the tyrosine residue phosphorylation, resulting in defects in insulin signaling (5).

Persistent hyperglycemia and dyslipidemia are the predominant factors of β-cell dysfunction, leading to excessive production of reactive oxygen species (ROS) and insulin resistance. ROS decreases insulin secretion from β-cells by reducing insulin gene expression by binding to several insulin gene promoter transcriptional factors. Treatment with antioxidants or overproduction of antioxidant enzymes can restore equilibrium in insulin production in patients with diabetes (6).

Plants and animals have been used for medicinal purposes by different medical disciplines since time immemorial. With the advent of science and demand for decreased mortality rate associated with the disorder, adverse effects of currently available medications and the need to improve quality of life demand further research to provide better regimens. At present, antidiabetic agents such as metformin obtained from *Galega officinalis* and acarbose from *Actinoplanes utahensi* both are of natural origin (7). *Ajuga iva, Moringa pergrina, Rhazya stricta, Cynomorium coccineum, Ducrosia anethifolia, etc*. are the plants used in the Middle East as antidiabetic agents (8–11).

Among plant divisions, the genus “*Brugmansia*” from the Solanaceae family is of prime importance due to its potential pharmacological benefits. The native use of this family is reported to have beneficial effects against inflammation, diabetes, rheumatic arthritis, body aches, etc. (12, 13). Flavonoids from *B. aurea* have previously been reported to possess antioxidant activity (14).

Although various parts of *the Brugmansia* genus are used traditionally, however, leaves and flowers are the most important parts for various aspects. They are recommended for external or oral use only if the preparation method follows crushing and decoction (15). The plants belonging to the genus *Brugmansia* have a wide range of uses, i.e., medical and non-medical, such as skin infections, various kinds of body pain, inflammation, cough, hallucination, and evil protection (16). The research presented in this article aimed to identify and report the potential antidiabetic effects of specie *B. aurea* by reducing insulin resistance in animals with the disease. Previous studies reported the anti-inflammatory (14, 17) and anticholinergic activities (18) of this plant. However, no studies are available on its antidiabetic activity. Therefore, the aim of this study was to identify and report the potential antidiabetic effects of *B. aurea* and elucidate the possible mechanism of action through *in vivo* and *ex vivo* experimental models.

## Materials and methods

### Collection and extraction of plants

The leaves of *B. aurea* were freeze dried and vacuumed for 1 day before being ground into a coarse powder in a mixer miller. The first stage in the extraction was to macerate 1:5 of powdered leaves in methanol for 3 days. The macerated powder was filtered initially through muslin cloth and then filtered paper. The filtered material was then passed through the rotary evaporator for solvent extraction (19). After maximum solvent extraction, the extra solvent was evaporated through an air drying process. The plant was identified and registered in the “Herbarium of medicinal plants, Comsats University Islamabad, Abbottabad campus, under the registration number ‘CUHA-185.”'

### Gas chromatography-mass spectroscopy and liquid chromatography-mass spectrometry analyses of *B. aurea* leaf extract

#### Gas chromatography-mass spectroscopy

Using the Chem-Station (Agilent Technologies) software, the gas chromatography-mass spectroscopy (GC-MS) control and data processing were done by comparing their mass spectra. The retention period, *m*/*z*, percentage of spectral similarity, and internal standard were all used to analyze metabolite identification using spectral matching (MS) using marketed libraries (NIST). The components' mass spectrum ranges were compared to the NIST and CAS libraries' compound registers. The names of the compounds were identified using their molecular weights, retention times, and concentrations.

#### Liquid chromatography-mass spectrometry

Crude extracts were analyzed using mass spectroscopy on an Agilent HPLC System 1290. An Agilent Zorbax XDB-C18 column with a narrow bore of 3.5 μm, a thickness, and a size of 2.1 × 150 mm, was used for reverse phase (RP) chromatography. The column temperature was maintained at 25°C. The UV-Vis variable UV detector used a variable wavelength of 220 nm for detection. A 0.5 ml/min elution gradient was used. Solvent A (0.1% formic acid in water) and Solvent B (0.1% formic acid in methanol) made up the elution gradient (MeOH). The gradient was initially established at 95% solvent A and 5% solvent B at 0 min. Then, over 25 min, it was linearly changed to 100% solvent B with 5% solvent A. For the following 5 min, this composition remained unchanged. A quadrupole (Time of Flight) TOF mass spectrometer was connected to the LC system (Agilent 6520 Accurate-Mass Q-TOF mass spectrometer with dual ESI source). The LCMS system captured real-time mass spectrometer data (spectrogram). The tuning parameters were set as shown in Table 1.

**Table 1:** Tuning parameter of liquid chromatography-mass spectrometry (LCMS).

**Mode**	**MS only**
Ion polarity	Positive
Vcap	4,000 V
Fragmentor voltage	125V
Skimmer	65 V
OCT 1 R*f*Vpp	750 V
Drying gas	10 L/min
Gas temperature	300°C
Nebulizer	45 psig
Mass range (*m*/*z*) Min	100
Max	3,200
Reference ions used	121.0508
	922.0097
Acquisition rate (spectra/s)	1.03
Acquisition time (ms/spectrum)	973
Transients/spectrum	9,632

### Experimental animals

For this study, Wistar rats (females) aged 4–6 months old with a weight of 150–200 g were selected. They were housed and caged in the animal house of the Riphah Institute of Pharmaceutical Sciences, RIU Lahore Campus, Lahore, Pakistan. All experiments and procedures were approved by the REC/RIPS-LHR (Research Ethical Committee of Riphah International University Lahore) under the authorization number of *Ref. No. REC/RIPS-LHR/2022053*. Room temperature and humidity were kept under control, i.e., 25°C ± 3 and 55%−70%, respectively.

### Induction of diabetes

All the groups except the control group were subjected to overnight fasting and then administered with alloxan (150 mg/kg) freshly mixed with 0.9% normal saline solution (20). These groups were provided with a 6% dextrose solution for 1 day to protect the animals from severe hypoglycemic conditions. The animals with blood glucose levels above 200 mg/dl were selected for further study (21).

### Study design

Animals were divided equally into nine groups (*n* = 6) for the treatment for 6 weeks orally on daily basis. Group I and II were normal healthy control and diabetic control (administered with alloxan 150 mg/kg), respectively. Group III was treated with pioglitazone at a dose level of 3.98 mg/kg. Group IV received metformin at 44.29 mg/kg. Group V was treated with glimepiride at 0.18 mg/kg. Group VI received an acarbose 8.86 mg/kg dose. Group VII, VII, and IX were treated with 100, 200, and 400 mg/kg *B. aurea* extract, respectively.

### Determination of weight variation and blood glucose

Body weight was recorded before the study and daily to calculate the dose for the individual animal. Data obtained were recorded for result evaluation. Blood glucose levels were checked weekly and on the day of dissection after overnight fasting at the end of the study (22).

### Oral glucose tolerance test (OGTT)

For this assessment, animals were kept for overnight fasting and injected with 2 g/kg of glucose orally, and blood glucose was measured at 0, 15, 30, 60, and 120 min by a one-touch glucometer (23).

### Insulin sensitivity measurement

Animals were injected with short-acting insulin at a dose of 0.5 IU/kg. Blood glucose levels were measured at 0, 15, 30, and 60 min by tail vein puncture. The data were obtained and assessed for the maximal hypoglycemic response after insulin injection (24).

### Biochemical assay

Liver enzymes, i.e., ALT, AST, and ALP, were measured using their appropriate kits (CC1223, CC1213, CC1016 by MTD Diagnostic Italy, respectively). Serum creatinine and urea levels were measured for evaluation of kidney function (CC1182, and CC1311 by MTD Diagnostic Italy, respectively). Serum cholesterol, LDL, HDL, and triglyceride levels were measured to assess the lipid profile (CC1132, CC1302 by MTD Diagnostic Italy, respectively) (23).

### Homogenization of the sample and estimation of antioxidants

Animals were euthanized and sacrificed after the study period. The pancreas, kidney, and liver were removed, washed with ice-cold water to remove any blood, and placed in phosphate buffer with a pH of 7.4. A tissue sample of 10% (w/v) for the estimation of antioxidant activity was prepared in a 0.1 M phosphate buffer with a pH of 7.4. This homogenate was centrifuged at 10,000 rpm for 15–20 min, and the supernatant was used to estimate antioxidant enzyme levels.

### Protein estimation

Lowry's method for protein estimation has two steps; first, the supernatant was mixed with an alkaline CuSO_4_ (copper sulfate) solution in the presence of 2% tartrate and kept for 10 min. In the second step, the Folin-Ciocalteu reagent was added to the solution and incubated for 30 min. Color enhancement by the reaction between tetra dentate copper and Folin-Ciocalteu reagent was checked, and absorption was measured at 750 nm (25).

The protein level was measured by using the following formula:


Y=0.00007571x+0.00004762

### Measurement of catalase level (CAT)

For catalase in a 3 ml cuvette, 0.05 ml of supernatant solution, 1.95 ml of phosphate buffer (50 mM), and 1 ml of hydrogen peroxide H_2_O_2_ (30 mM) were mixed. The change in absorbance was measured at 240 nm every 30 sec after a 15-s interval. The extinction coefficient in mM (millimoles) of H_2_O_2_ was used to measure catalase activity, and results were calculated in μM (micromoles) of oxidized H_2_O_2_. The catalase activity was expressed as μmole/min/mg of proteins (26).


$$CAT\;activity\;=\;\frac{\delta O.D}{E\times Sample\;volume\;(ml)\times Proteins\;(mg)}$$

*E* = *extinction coefficient* of hydrogen peroxide (mmol/cm); δO.D = changes in absorption per minute.

### Measurement of superoxide dismutase level

For superoxide dismutase level (SOD) measurement, 0.1 ml of supernatant solution and EDTA (1 × 10^−4^) were mixed with 1 ml of 1 mM epinephrine and 0.5 ml of carbonate buffer with a pH of 9.7. The absorption was measured for 3 min at 480 nm. The unit of enzyme activity was written as U/min/mg (27).

The protein level was measured for SOD activity from the following regression equation.


Y=0.0095x+0.1939

### Measurement of malondialdehyde

For this purpose, 0.5 ml of tissue homogenate mixed with 2.5 ml of TCA (trichloro acetic acid) 10% w/v were centrifuged for 10 min at 1,000 rpm, kept in a water bath for 15 min, and then cooled down to room temperature. Moreover, 2 ml of the reaction mixture was transferred to the test tube containing 1 ml of TBA (0.67% w/v) and again placed in the water bath. Absorbance was checked at 532 nm (28).


$$\begin{array}{l} Conc.\;of\;MDA=\text{ OD532 }\times \;100\;\times \text{ Tv}/(1.56\;\times \;105)\\ \;\times \text{ d }WT\;\times \;Av \end{array}$$

OD = absorbance at 532 nm, Tv = total volume (4 ml), d WT = weight of disserved tissue (1 g), Av = aliquot volume (1 ml), (1.56 × 105) is the molar extinction coefficient.

### Measurement of glutathione level

For estimation of reduced glutathione (GSH), 1 ml of 10% TCA and supernatant solution was mixed and precipitated. This precipitate was combined with 4 ml of phosphate solution and 0.5 ml of DTNB (dithio-bis-nitrobenzoic acid) reagent. Absorption of the mixture was measured at 412 nm. The values of absorption were written as nM of GSH per milligram of protein (28).


$$Reduced\;Glutathione\;(GSH)\;level\;=\;\frac{Y-0.00134}{0.0134\times \frac{D_{F}}{T\times VU}}$$

Y = Absorption at 412 nm; D_F_ = Dilution factor (1); T = Tissue homogenate; VU = aliquot volume.

### Determination of HbA1c, insulin, and insulin resistance

The day before dissection, animals were fasted overnight and euthanized with isoflurane 3–5% diluted with oxygen. Fresh blood was collected by heart puncture for HbA1c performance. The serum was subjected to measuring insulin levels by an ELISA kit (CK-E30973 by Zokeyo Biological Co., Ltd., China). Insulin resistance (IR) was calculated using the HOMA2 calculator (https://www.dtu.ox.ac.uk/homacalculator/) (23, 24, 29).

### Histopathological examination

The pancreas, kidney, and liver were removed and washed with ice-cold water. The tissue samples were stabilized in a 10% formalin solution and kept embedded in paraffin wax. After fixation, these samples were cut into 5 μm section thickness. These sections were stained with hematoxylin and eosin. After staining, they were examined for histopathological changes (28).

### Statistical analysis

All values were statistically analyzed as mean ± SEM. Evaluation of data performed by one and two-way ANOVA. Values were plotted on Graph-Pad prism (San Diego, CA) software with a very high significance (*p* < 0.001), moderate significance (*p* < 0.01), and mild significance (*p* < 0.05) level.

## Results

### Effect on weight

**Figure 1:**
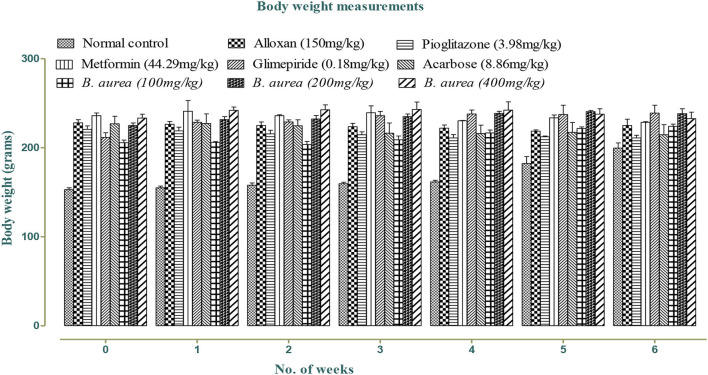
Effect of different treatments on body weight in alloxan-induced diabetes.

Weight was reduced in all standards and *B. aurea* dosing groups, but no significant reduction was observed in either of the groups, as shown in Figure 1 below:

### Effect on blood glucose

**Figure 2:**
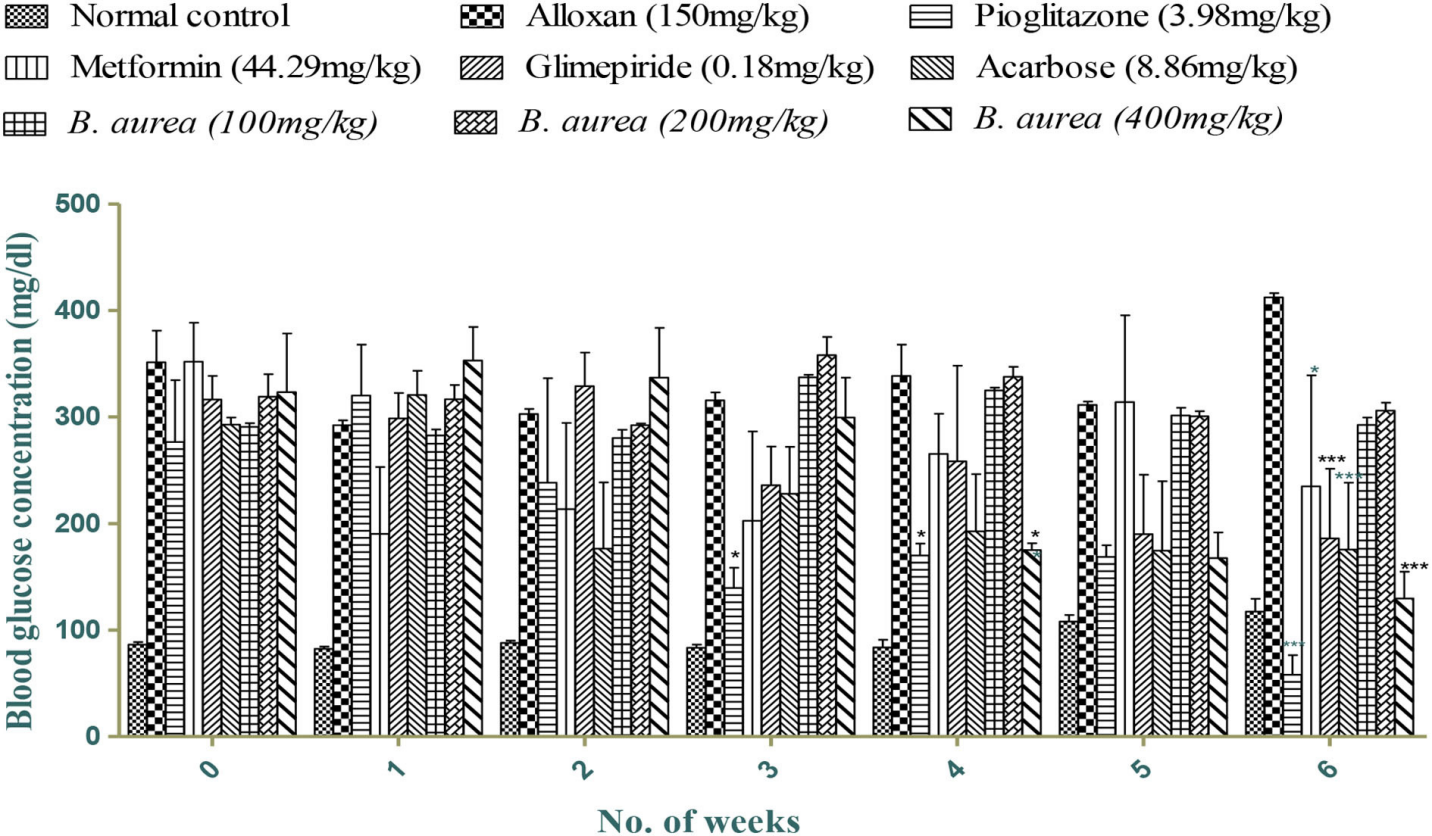
Effect of treatments on blood glucose concentrations (mg/dL). ***p < 0.001, **p < 0.01, *p < 0.05 significant values were obtained after comparison with the diseased control.

Figure 2 shows mild to a highly significant reduction (*p* < 0.05 to *p* < 0.001) in blood glucose levels with all standards and *B. aurea* (400 mg/kg), while no significant results were obtained with *B. aurea* at a dose of 100 and 200 mg/kg. Significant reduction in glucose levels was observed from the third week of treatments in which only pioglitazone showed a significant reduction in comparison to the diseased control group. While *B. aurea* at a dose level of 400 mg/kg showed a significant reduction in glucose level from the fourth week of treatments and continued till the end of the study compared with the diseased group.

### Effect of OGTT

**Figure 3:**
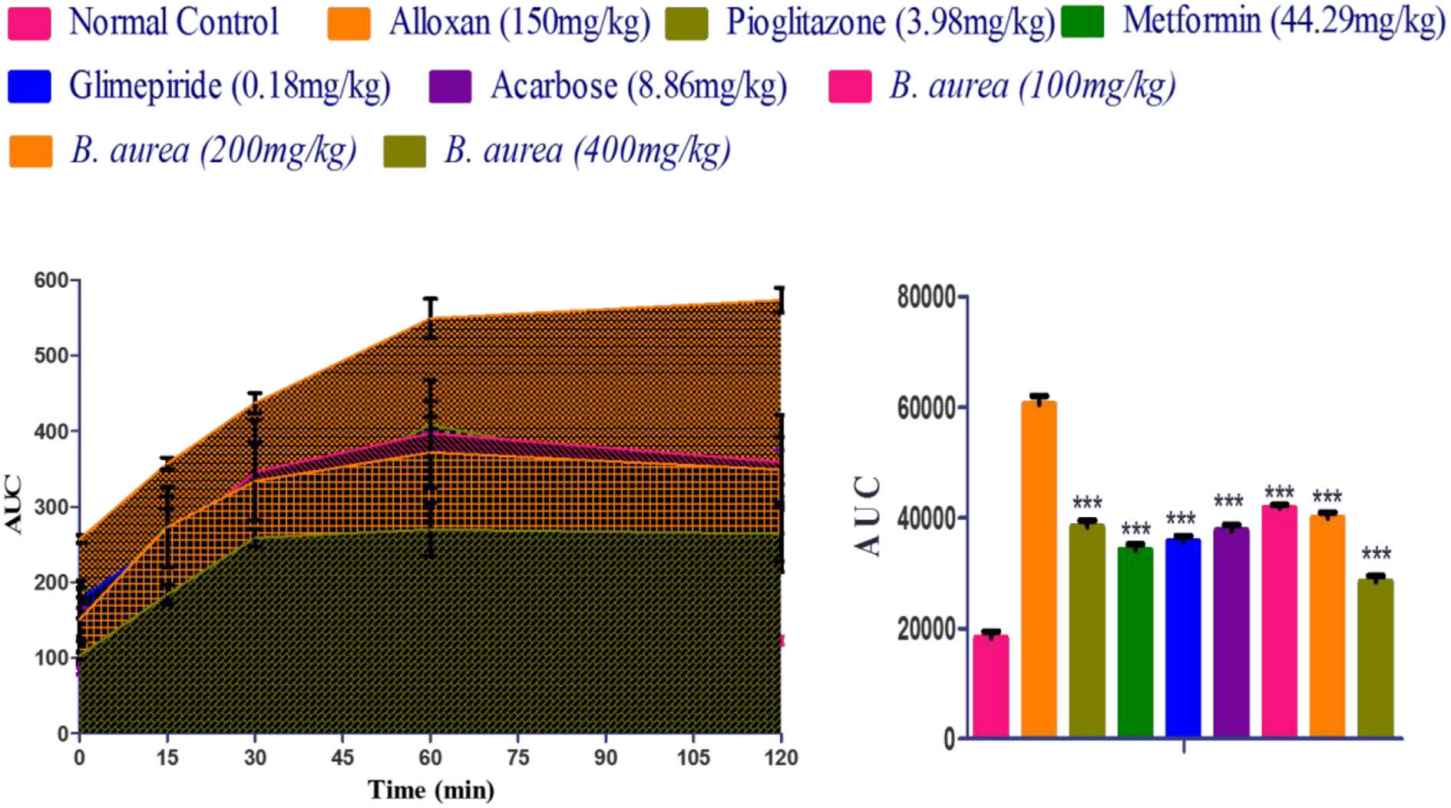
Measurement of the area under the curve (AUC) of the treated groups that depicted the findings of the oral glucose tolerance test. ***p < 0.001, **p < 0.01, *p < 0.05 significant values were obtained after analysis by one-way ANOVA.

Comparison with the diseased control shows significantly decreased (*p* < 0.001) blood glucose concentrations in all standards and *B. aurea* treated groups. The results of AUC (area under the curve) indicated that *B. aurea* at all dose levels and standard oral hypoglycemic agents lower the glucose concentration to a considerable extent compared with the diabetic control group, as shown by the graph below in Figure 3.

### Insulin sensitivity measurement

**Figure 4:**
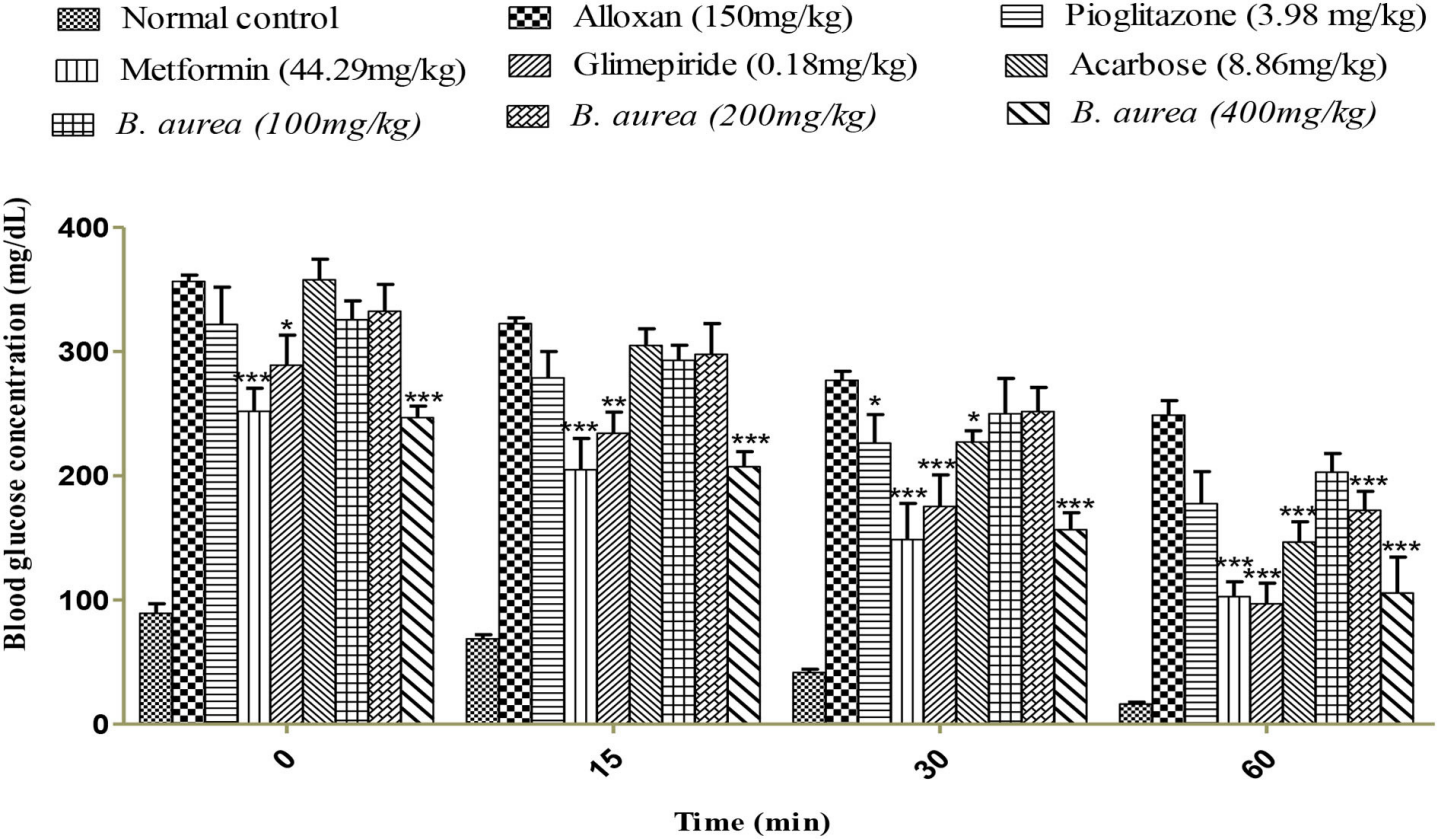
Effect of a 0.5 IU/kg insulin injection on blood glucose concentrations (mg/dl). ***p < 0.001, **p < 0.01, *p < 0.05 significant values were obtained after comparison with the diseased control.

Animals that received metformin and *B. aurea* 400 mg/kg showed a highly significant reduction (*p* < 0.001) in blood glucose levels. Other standards represented mild (pioglitazone *p* < 0.05) to moderately (Acarbose and glimepiride *p* < 0.01) significant reductions. The treatment group *B. aurea* 100 mg/kg showed no significant results, while *B. aurea* 200 mg/kg showed a highly significant reduction (*p* < 0.001) at the end of the study, as shown in Figure 4 below.

### Effect on lipid profile

All standard groups showed a highly significant decline (*p* < 0.001) in serum TC levels, while only the pioglitazone and metformin standards were found effective in reducing serum TG levels efficiently. Some of the standard groups increased serum HDL levels from mild (*p* < 0.05) to high (*p* < 0.001) significant levels. *B. aurea* at all doses lowered serum TC and TG levels significantly (*p* < 0.001) compared to the diseased group. Only *B. aurea* at a dose of 400 mg/kg was found effective in significantly increasing (*p* < 0.05) serum HDL, as mentioned in Table 2.

**Table 2:** Effect of *B. aurea* and standard treatments on the lipid profile of diabetic rats.

**Group**	**TC (mg/dl)**	**TG (mg/dl)**	**LDL (mg/dl)**	**HDL (mg/dl)**
Normal control	57.33 ± 3.28	106.33 ± 2.60	36.67 ± 1.20	74.33 ± 2.03
Alloxan (150 mg/kg)	232.67 ± 23.68	268.00 ± 28.54	86.33 ± 4.26	35.33 ± 2.33
Pioglitazone (3.98 mg/kg)	33.33 ± 8.41^***^	199.33 ± 6.39^***^	75.00 ± 2.03	56.67 ± 1.45
Metformin (44.29 mg/kg)	17.33 ± 2.73^***^	223.33 ± 3.28^***^	70.67 ± 2.03	70.00 ± 3.79^**^
Glimepiride (0.18 mg/kg)	141.33 ± 1.45^***^	276.33 ± 12.45	77.67 ± 3.53	67.00 ± 6.66^*^
Acarbose (8.86 mg/kg)	148.00 ± 4.58^***^	296.33 ± 10.09	85.67 ± 6.49	78.67 ± 5.36^***^
*B. aurea* (100 mg/kg)	123.00 ± 6.66^***^	125.67 ± 2.91^***^	43.00 ± 2.31^***^	54.00 ± 6.56
*B. aurea* (200 mg/kg)	148.00 ± 1.73^***^	152.67 ± 3.38^***^	66.33 ± 4.81	56.67 ± 2.33
*B. aurea* (400 mg/kg)	126.33 ± 5.93^***^	179.33 ± 4.91^***^	64.33 ± 2.40	66.00 ± 3.21^*^

TC, total cholesterol; TG, total triglycerides; LDL, low-density lipoproteins; HDL, high-density lipoproteins.

Data presented as (n = 6) mean ± SEM.

*** p < 0.001.

** p < 0.01.

* p < 0.05 significant values obtained after comparison with the diseased group.

### Assessment of liver and renal function

Upon statistical analysis, all standard treatments had highly significant reducing (*p* < 0.001) capability on serum AST, while only a few standards possessed mild significant declining (*p* < 0.05) capacity on serum ALT levels. *Brugmansia aurea* on all doses had a highly significant reduction (*p* < 0.001) in serum AST level compared to the standard treatments. Table 3 showed all standard oral hypoglycemic agents of different classes, and *B. aurea* at all doses had a highly significant lowering capacity (*p* < 0.001) on the urea level compared with the diseased group.

**Table 3:** Effect of *B. aurea* and standard treatments on LFT and RFT in diabetic rats.

**Group**	**AST (IU/l)**	**ALT (IU/L)**	**Uric acid (mg/dl)**	**Urea (mg/dl)**
Normal control	406.00 ± 4.04	142.67 ± 3.28	1.25 ± 0.02	42.33 ± 0.88
Alloxan (150 mg/kg)	814.00 ± 38.37	209.00 ± 1.15	8.23 ± 0.38	136.77 ± 1.36
Pioglitazone (3.98 mg/kg)	266.67 ± 3.18^***^	157.67 ± 0.88	0.81 ± 0.03	84.83 ± 6.91^***^
Metformin (44.29 mg/kg)	154.67 ± 5.78^***^	151.00 ± 5.51^*^	0.72 ± 0.01	66.33 ± 2.33^***^
Glimepiride (0.18 mg/kg)	165.67 ± 4.48^***^	148.33 ± 8.84^*^	0.71 ± 0.02	75.13 ± 1.05^***^
Acarbose (8.86 mg/kg)	184.67 ± 6.98^***^	172.33 ± 4.91	0.79 ± 0.01	90.50 ± 4.79^***^
*B. aurea* (100 mg/kg)	300.33 ± 6.36^***^	188.33 ± 20.58	1.13 ± 0.08	109.67 ± 11.21^***^
*B. aurea (200 mg/kg)*	284.33 ± 11.72^***^	182.33 ± 7.69	1.14 ± 0.09	112.80 ± 2.41^***^
*B. aurea (400 mg/kg)*	192.67 ± 27.88^***^	166.33 ± 4.06	0.72 ± 0.02	87.20 ± 6.57^***^

AST, aspartate aminotransferase; ALT, alanine aminotransferase.

Data presented as (n = 6) mean ± SEM.

*** p < 0.001.

** p < 0.01.

* p < 0.05 significant values were obtained after comparison with the diseased group.

### Effect on antioxidant enzymes

#### Pancreas

**Figure 5:**
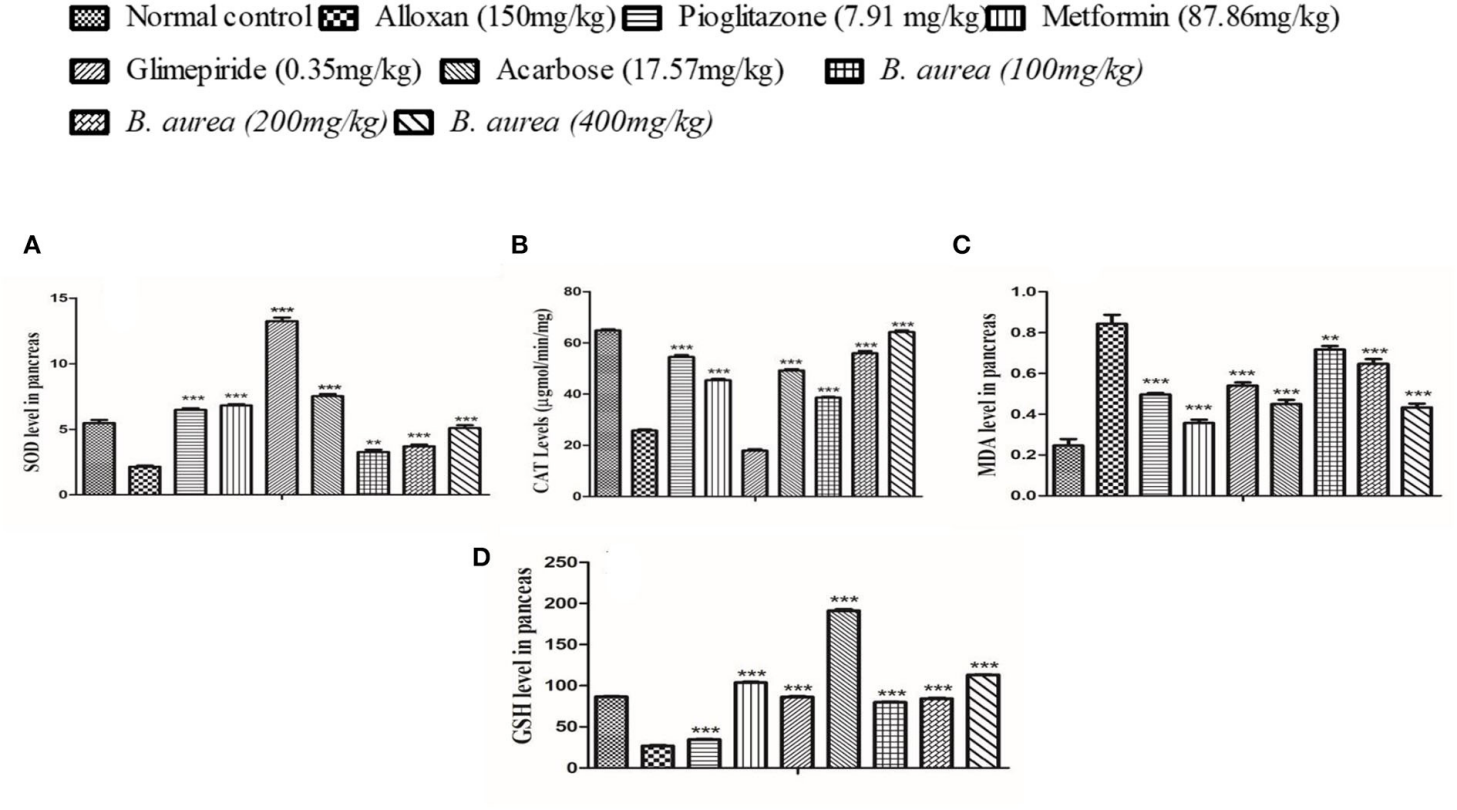
Effect of *B. aurea* on antioxidant enzyme levels in the pancreas of diabetic animals. **(A)** superoxide dismutase (SOD), **(B)** catalase (CAT), **(C)** melanoaldehyde (MDA), and **(D)** reduced glutathione (GSH). ***p < 0.001, **p < 0.01, *p < 0.05 significant values.

Superoxide Dismutase, ALT, malondialdehyde (MDA), and GSH levels in the pancreas of rats were significantly increased (*p* < 0.001) in groups of standard drugs compared to the diseased control group. *Brugmansia aurea* at all dosing concentrations possessed a moderate to highly significant increase (*p* < 0.01 to *p* < 0.001) in all antioxidant enzymes, suggesting a protective effect of the plant extract against diabetic complications, as shown in Figure 5.

#### Kidney

**Figure 6:**
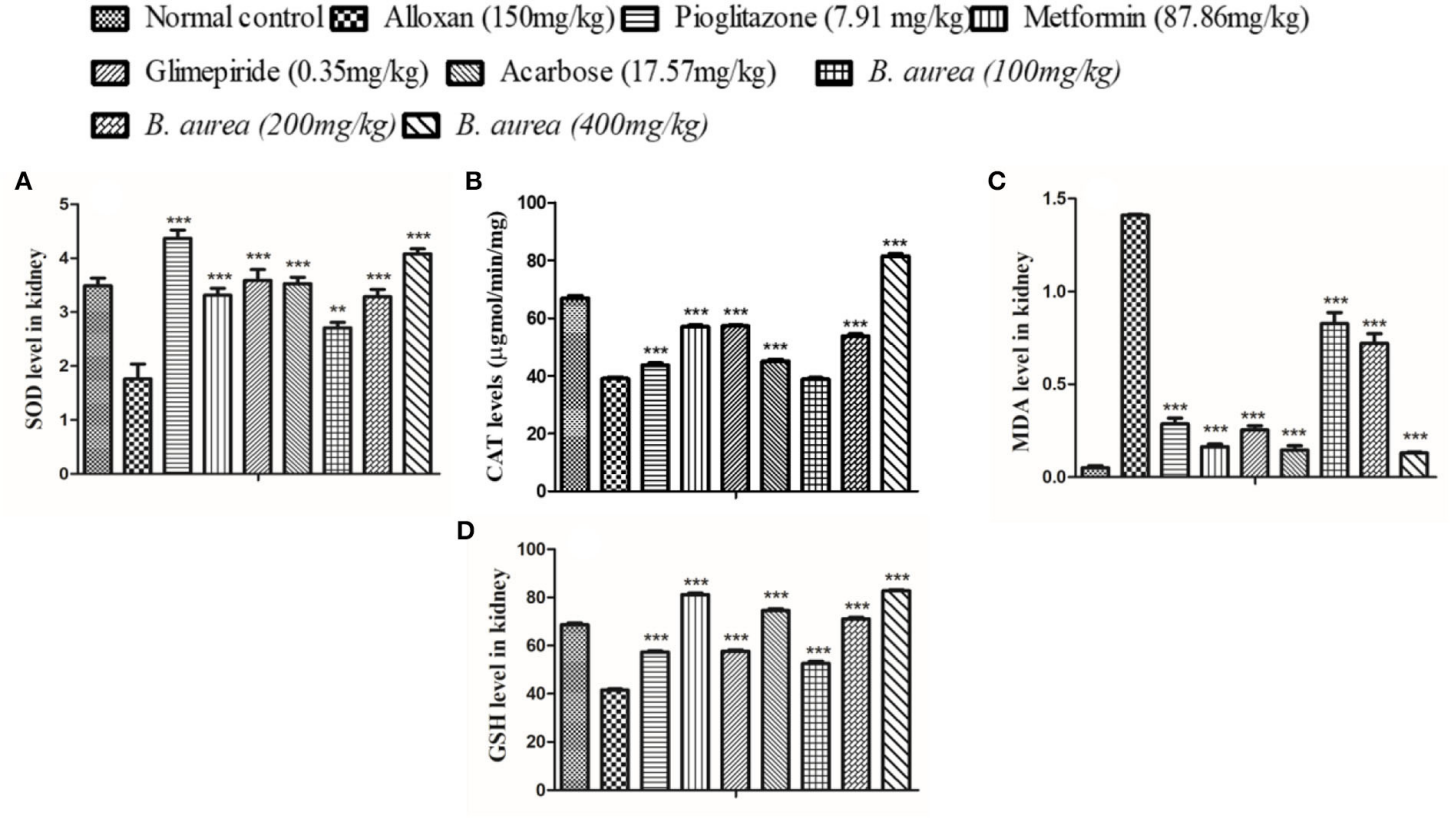
Effect of *B. aurea* on antioxidant enzyme level in the kidney of diabetic animals. **(A)** superoxide dismutase (SOD), **(B)** catalase (CAT), **(C)** melanoaldehyde (MDA), and **(D)** reduced glutathione (GSH). ***p < 0.001, **p < 0.01, *p < 0.05 significant values.

All antioxidant enzymes were significantly higher (*p* < 0.001) in standard and treatment groups, while MDA levels were lowered (*p* < 0.001) in all groups upon comparison with the diseased (Figure 6).

#### Liver

**Figure 7:**
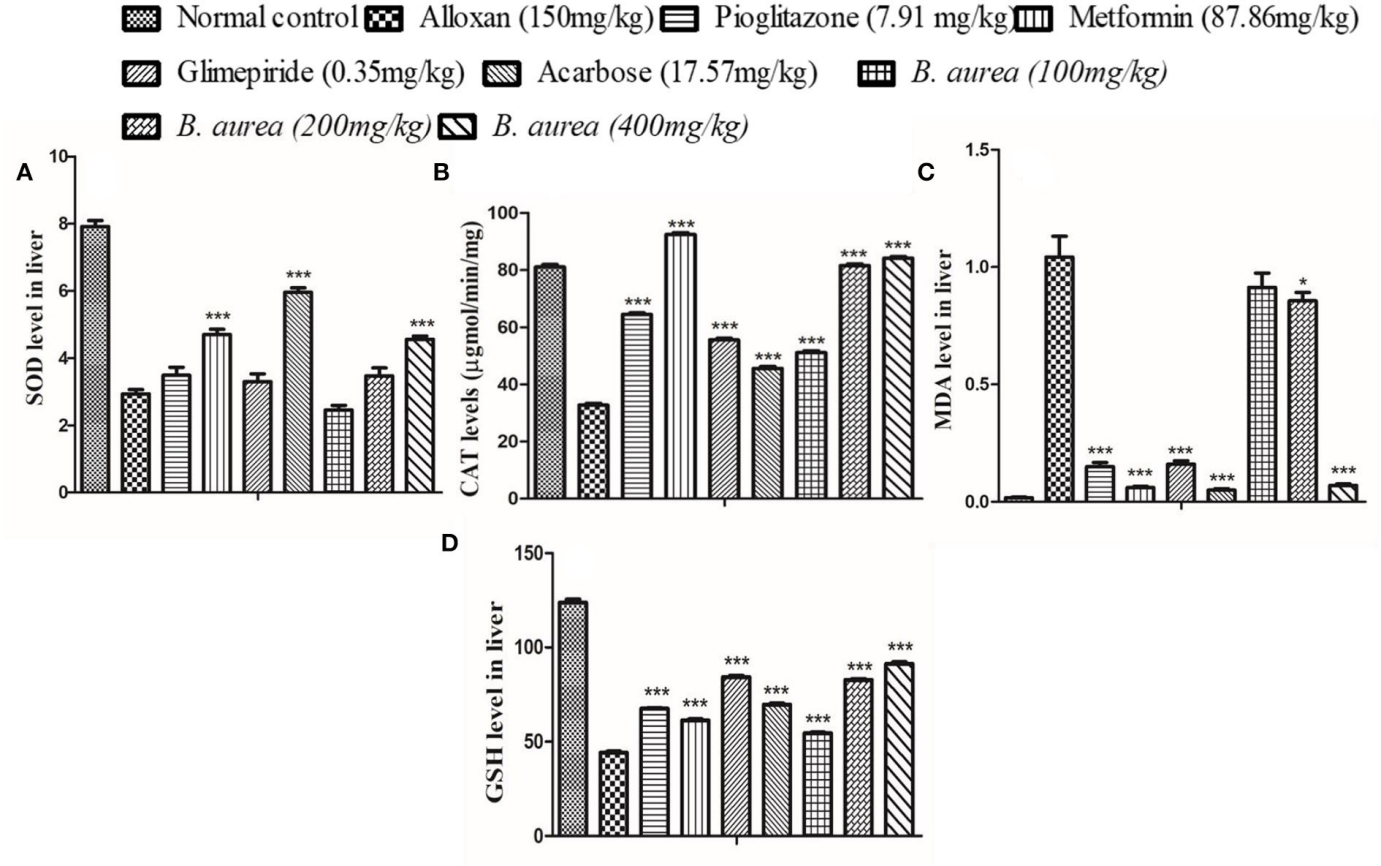
Effect of *B. aurea* on antioxidant enzyme level in the liver of diabetic animals. **(A)** superoxide dismutase (SOD), **(B)** catalase (CAT), **(C)** melanoaldehyde (MDA), and **(D)** reduced Glutathione (GSH). ***p < 0.001, **p < 0.01, *p < 0.05 significant values.

The liver of diabetic rats administered with metformin and Acarbose showed a highly significant increase (*p* < 0.001) in SOD, while other antioxidant defense enzymes presented a comparable increase/decrease with all oral hypoglycemics. *Brugmansia aurea* at a dose of 400 mg/kg produced similar effects as other standard oral hypoglycemic agents (Figure 7).

### Effect on HbA1c, insulin, and insulin resistance

**Figure 8:**
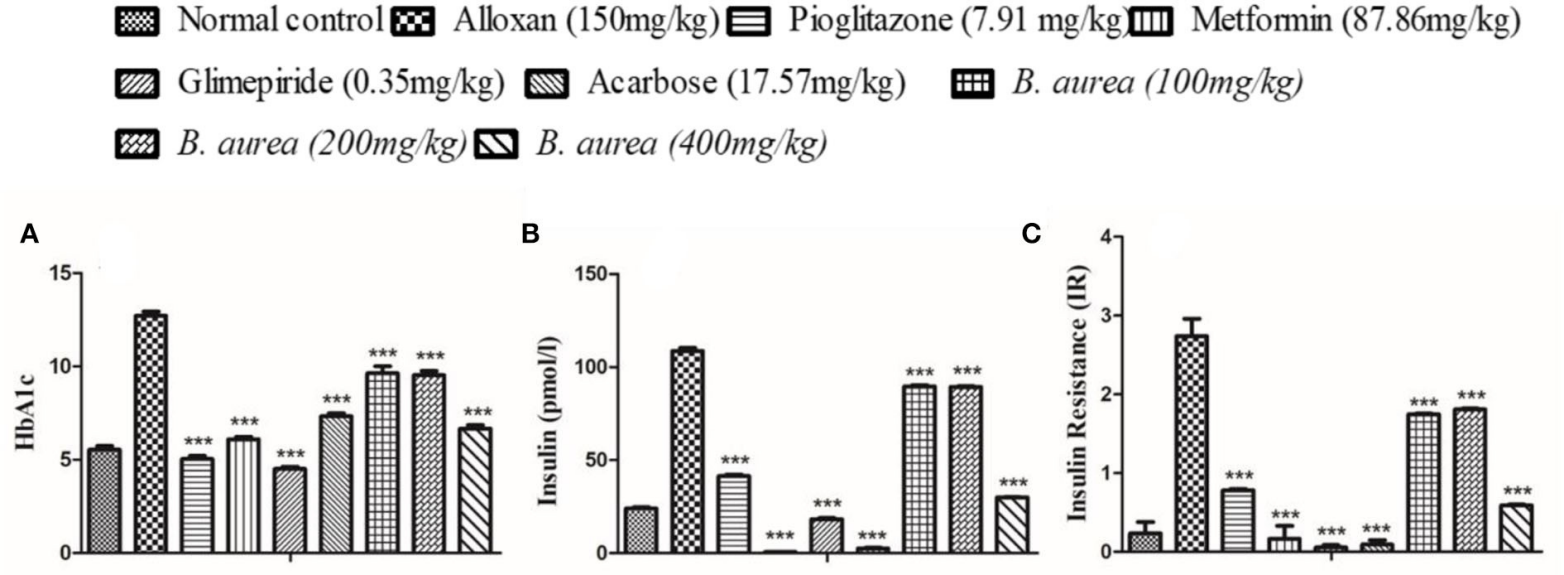
Effect of *B. aurea* on HbA1c, insulin, and insulin resistance in diabetic animals. **(A)** HbA1c, **(B)** insulin level, and **(C)** insulin resistance. ***p < 0.001, **p < 0.01, *p < 0.05 significant values.

All groups showed a similar, highly significant (*p* < 0.001) decrease in HbA1c level. The disease condition increased the level of insulin which was reduced to a significant level (*p* < 0.001) by the standard drugs and plant extract. Upon calculation, it was observed that all standards and *B. aurea* doses lowered insulin resistance significantly (*p* < 0.001) compared to the alloxan group, as presented in Figure 8.

### Histopathological examination

**Figure 9:**
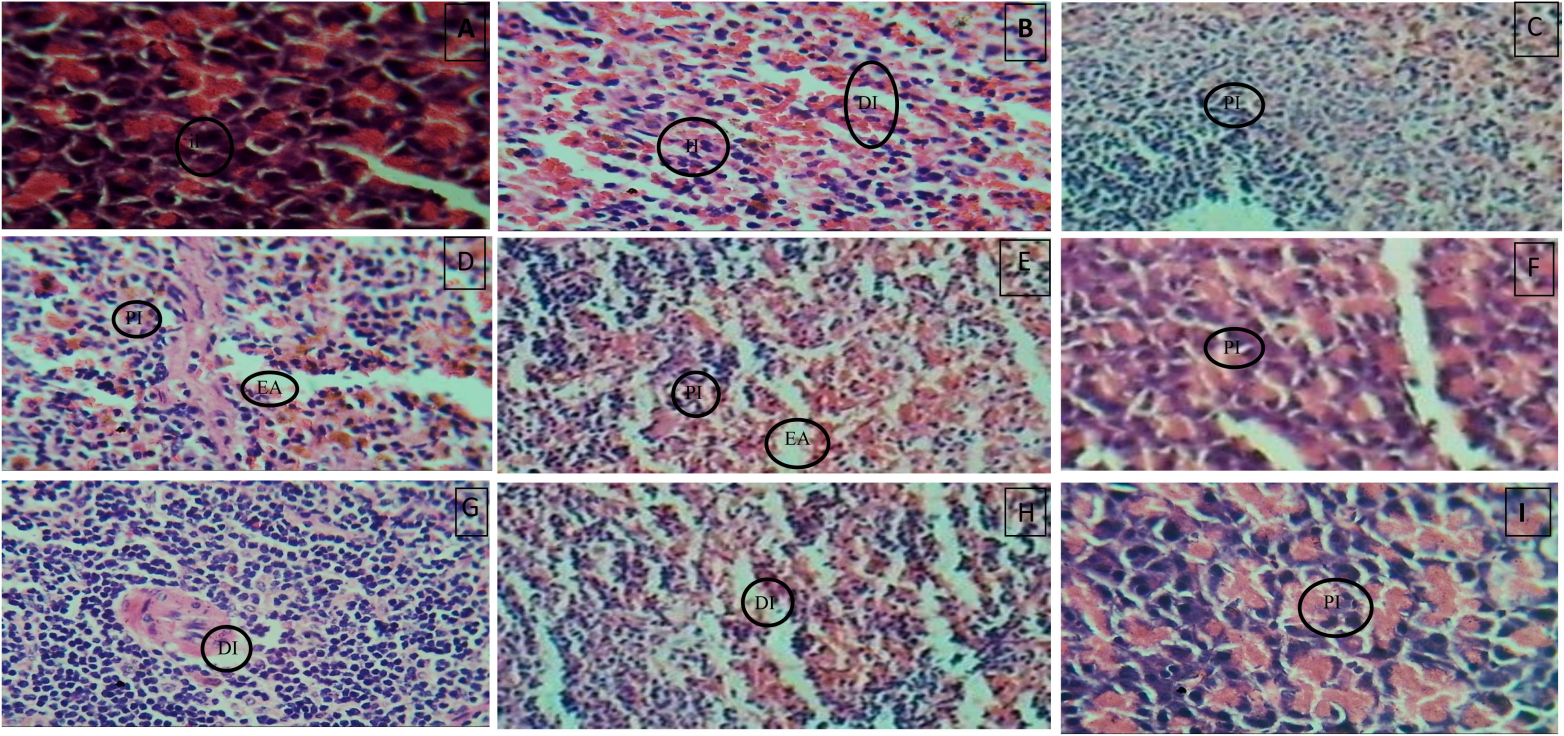
**(A)** Normal control, **(B)** Diseased, **(C)** Pioglitazone 3.98 mg/kg, **(D)** Metformin 44.29 mg/kg, **(E)** Glimepiride 0.18 mg/kg, **(F)** Acarbose 8.86 mg/kg, **(G)** *B. aurea* 100mg/kg, **(H)** *B. aurea* 200 mg/kg, and **(I)** *B. aurea* 400 mg/kg. Note: IL, Islets of Langerhans; DI, Depleted islets; H, Hemorrhage; PI, Preserved islets; EA, Exocrine acini.

**Figure 10:**
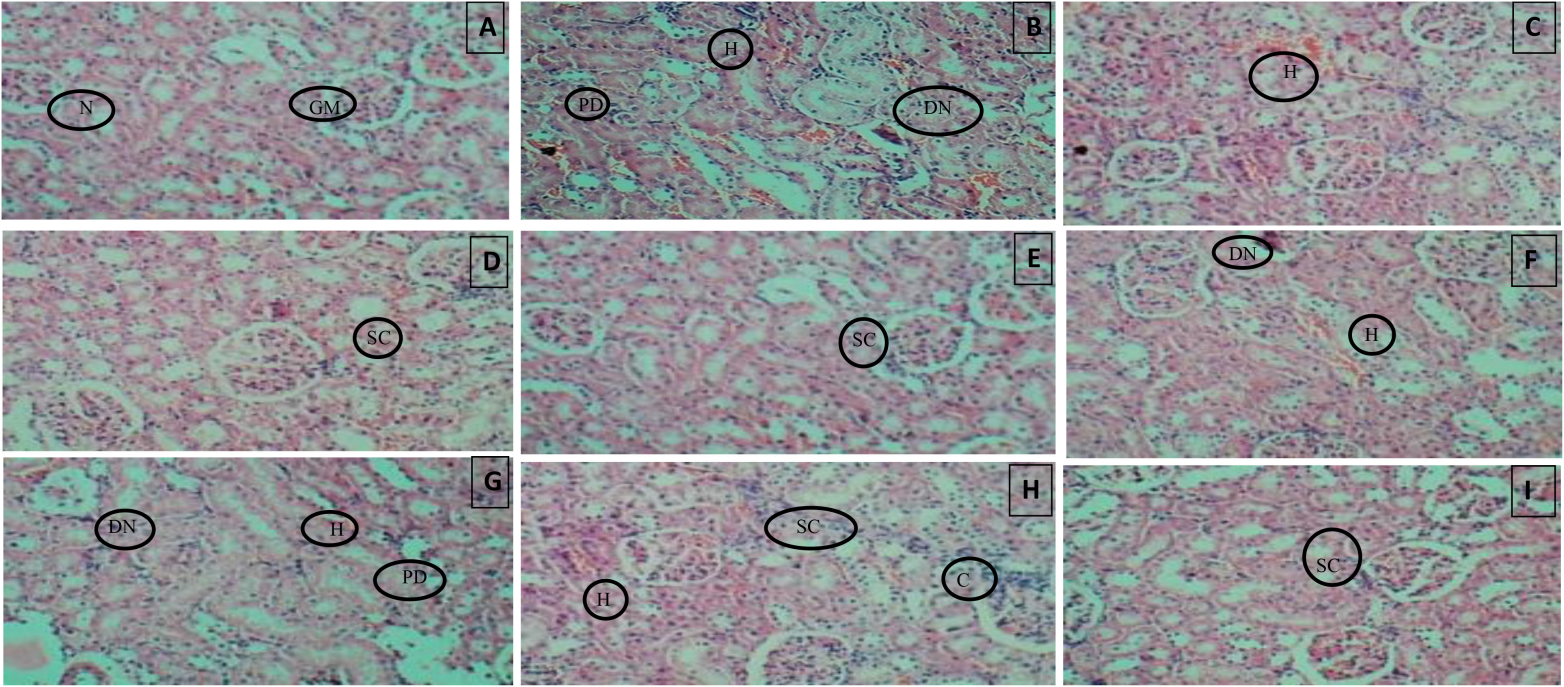
**(A)** Normal control, **(B)** Diseased, **(C)** Pioglitazone 3.98 mg/kg, **(D)** Metformin 44.29 mg/kg, **(E)** Glimepiride 0.18 mg/kg, **(F)** Acarbose 8.86 mg/kg, **(G)** *B. aurea* 100 mg/kg, **(H)** *B. aurea* 200 mg/kg, and **(I)** *B. aurea* 400 mg/kg. Note: N, Nephron; GM, Glomerulus; P, Proximal tubule; PD, Proximal tubule damage; H, Hemorrhage; DN, Denatured nephron; SC, Suffused capillaries; C, Congestion of capillaries at Bowman's capsule.

**Figure 11:**
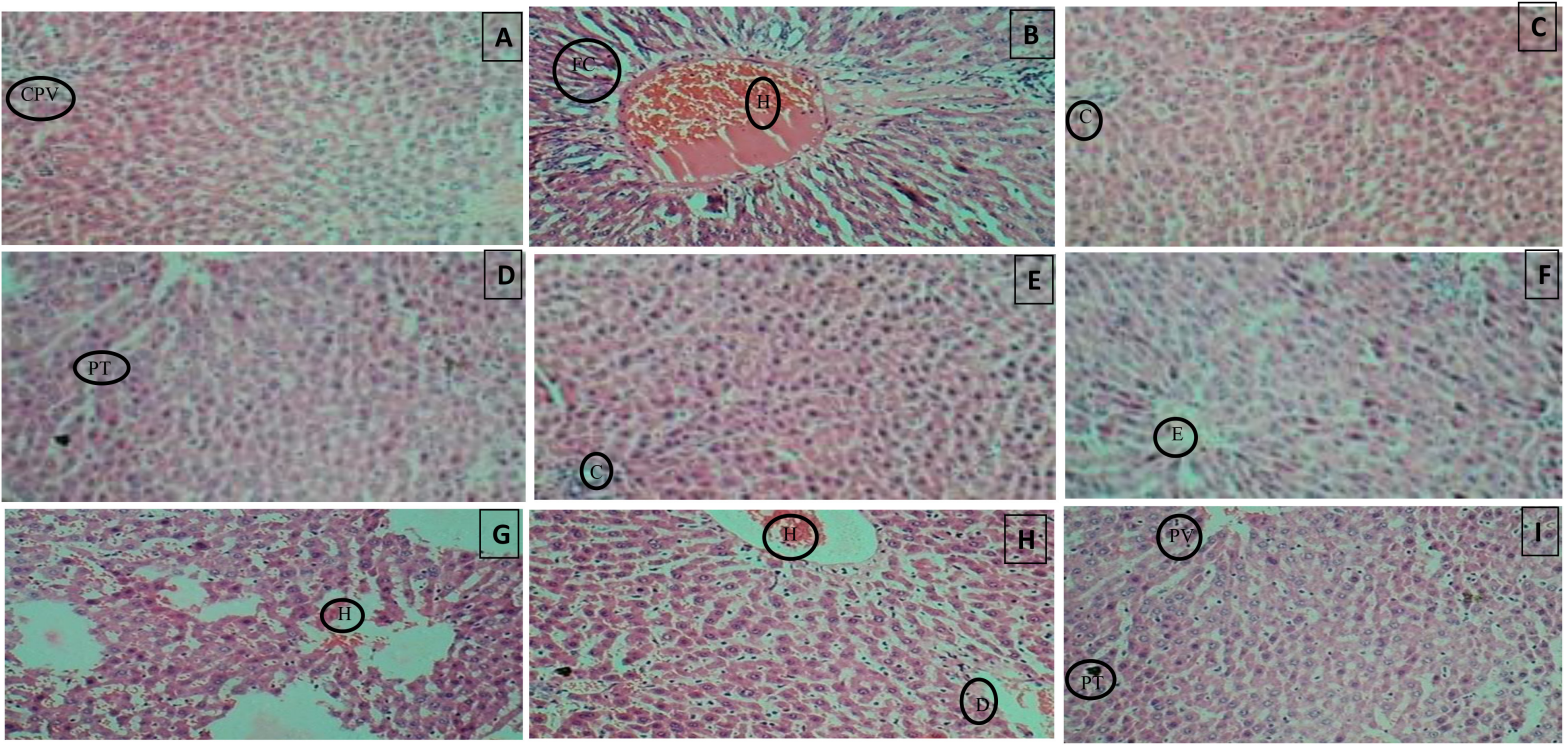
**(A)** Normal control, **(B)** Diseased, **(C)** Pioglitazone 3.98 mg/kg, **(D)** Metformin 44.29 mg/kg, **(E)** Glimepiride 0.18 mg/kg, **(F)** Acarbose 8.86 mg/kg, **(G)** *B. aurea* 100 mg/kg, **(H)** *B. aurea* 200 mg/kg, and **(I)** *B. aurea* 400 mg/kg. Note: CPV, Central portal vein; FC, Fat cells; H, Hemorrhage; C, Congestion; PT, Portal tract; E, Edema; D, Dilation.

Histopathology of diabetic rat pancreas, kidney and liver were shown in Figures 9–11.

### GCMS results

Gas chromatography-MS analysis revealed the presence of seven phytochemical components in the methanol leaf extract of *B. aurea*. The major compounds are hexadecanoic acid and methyl ester.

**Figure 12:**
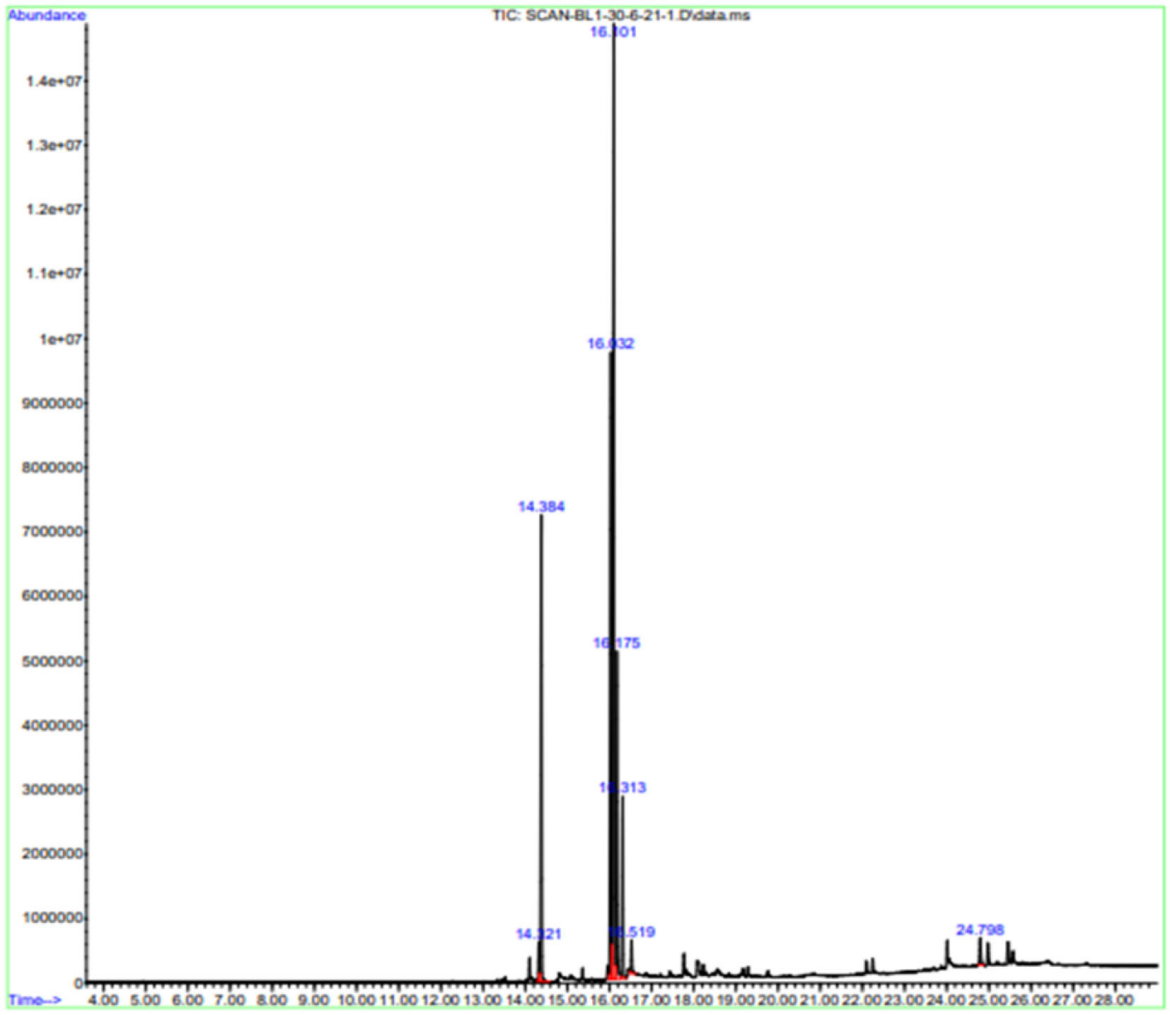
Chromatogram of leaf extracts of *B. aurea* analyzed by the developed GCMS method.

9,12-Octadecadienoic acid (Z, Z)-, methyl ester, 9,12,15-Octadecatrienoic acid, methyl ester, Phytol, Methyl stearate, and campestral with a percentage area of 15.54, 25.79, 37.52, 11.51, 5.73, 1.3, and 1.28, respectively (Table 4, Figure 12).

**Table 4:** Identified compounds in *B. aurea* leaves by the GCMS method.

**S. No**	**Compound**	**Percentage area**	**NIST/CAS**	**M. wt**	**RT**
1	Hexadecanoic acid, methyl ester	15.54	112–39–0	270.45	14.384
2	9,12-Octadecadienoic acid (Z, Z)-, methyl ester	25.79	112–63–0	294.47	16.023
3	9,12,15-Octadecatrienoic acid, methyl ester	37.52	7361–80–0	292.46	16.101
4	Phytol	11.51	150–86–7	296.53	16.175
5	Methyl stearate	5.73	112–61–8	298.5038	16.313
6	Neophytadiene	1.3	504–96–1	278.52	16.519
7	Campesterol	1.28	474–62–4	400.6801	24.798

### Liquid chromatography-mass spectrometry

**Figure 13:**
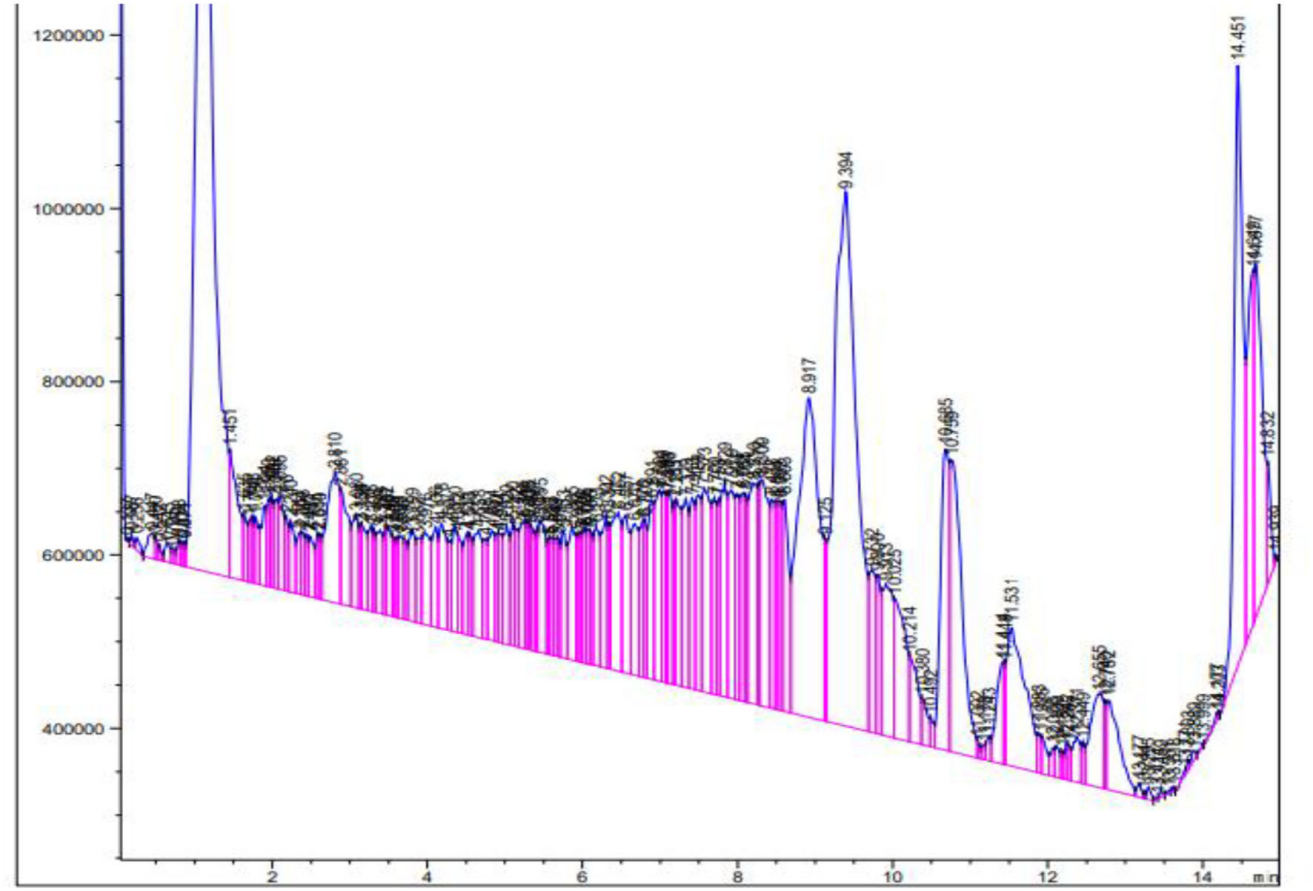
Chromatogram of flower extracts from *B. aurea* analyzed by the LCMS method.

The extract from *B. aurea* was analyzed through liquid chromatography-mass spectrometry (LCMS), as shown in Figure 13. The MS spectrum show peak with time and *m*/*z* value were analyzed one by one with the standard NIST library. Different compounds were observed when compared with the NIST library. Table 5 shows the retention time, molecular weight, and mass area of all the compounds. The compound that is present in maximum quantity is 2-(2-Carbomethoxyphenylamino)-4-phenyl-6-(4-phenoxyphenyl) pyrimidine with a mass area of 925,244, while the compound that is present at minimum concentration is Tetracosanoic acid, pentyl ester with a mass area of 334,551 in the case of a flower. However, in the case of leaves, the compound present in maximum quantity is Dihydromorphine, O, O'-bis(heptafluorobutyryl)- with a mass area of 29,645. Where are these data for leaves?

**Table 5:** Identified compounds in *B. aurea* flower extract by the LCMS method.

**S. No**	**Name of compound**	**Retention time**	**Molecular weight**	**Mass area**
1	1(2H)-Quinolinecarboxylic acid, 2-ethoxy-, 2-methylpropyl ester	1.13	317.20	16,017,229
2	Thieno[2,3-b]pyridine-2-carboxamide, 3-amino-4,6-dimethyl-N,N-di(2-propenyl)-	1.13	317.20	16,017,229
3	Adipic acid, eicosyl 2-methylpent-3-yl ester	1.451	803.60	1,087,640
4	3-Phenylacrylic acid, 5,6,7-triacetoxy-1-(3,4,5-triacetoxy-6 acetoxymethyltetrahydropyran-2-yloxy)-1,4a,5,6,7,7a-hexahydrocyclopenta[c]pyran	2.81	803.60	1,757,443
5	2-(2-Carbomethoxyphenylamino)-4-phenyl-6-(4-phenoxyphenyl) pyrimidin	2.881	804.60	925,244
6	Cyclohexanecarboxamide, N-benzyl-N-propyl	8.917	792.60	7,104,056
7	Tetracosanoic acid, pentyl ester	9.125	803.60	33,455
8	Phthalic acid, 2-methoxyethyl nonyl ester	9.125	803.60	334,551
9	(3,3-Dimethyl-1-butene)[bis(dicyclohexylphosphino)ethane]platinum(0)	9.394	701.50	12,493,676
10	Hexadecanoic.4-nitrophenyl ester	10.685	392.25	2,604,970
11	Fumaric acid,di(2-methylpent-3yl)ester	10.759	392.30	3,663,608
12	Fenoterol.5TMS derivative	11.414	663.45	722,664
13	Tries(2,3-dibromopropyl)isocyanurate	11.531	723.55	2,680,692
14	Isoquinoline,1-(3,5-dimethoxybenzyl)-1,2,3,4-tetrahydro-6-methoxy-	12.782	392.30	1,184,129
15	Rel-(2S.4S.10R)-2-[4-(2-P-bromophenyl)-2-oxoethyloxy)cinnamyloxy]-4-[3,4-(2-p-bromophenyl-2-oxo-ethyloxy)cinnamyloxy-4-methoxyphenyl]quinolizidine	14.451	830.60	5,402,115
16	PentanediamideN.N.N',N'-tetrabutyl-3-[(dibutylamino)carbonyl]-	14.832	793.60	435,667
17	Oxalic acid, monomorpholide, propyl ester	14.677	814.60	3,017,702

## Discussion

Diabetes mellitus is a chronic metabolic disorder growing immensely all over the globe due to various genetic, environmental, and lifestyle changes. These changes caused deterioration in insulin secretion or insulin resistance, leading to several micro and macrovascular abnormalities (1, 30, 31). According to the previous reports presented by Kandimalla et al., (32) and Kim et al., (2017), plants belonging to the Solanaceae family were useful against diabetes and oxidative stress, respectively (32, 33). Therefore, this study was conducted to find the potential benefits of *B. aurea* in the management of hyperglycemia and insulin resistance during the disease.

Standard oral hypoglycemic agents tend to manage hyperglycemia and other diabetic complications through various mechanisms, i.e., decreasing insulin resistance by acting on peroxisome proliferator-activated receptors (PPARs), making insulin more effective or releasing bound insulin, reducing hepatic gluconeogenesis and degradation of endogenous insulin (34, 35). Multiple standards from different groups were also selected in this study to find out the possible mechanism of action of our selected plant against diabetes. The crude extract of *B. aurea* at a dose level of 400 mg/kg exhibited identical significant (*p* < 0.001) effects in reducing blood glucose levels as standard oral hypoglycemic metformin, confirming its potential benefits during diabetes.

After OGTT performance, it was observed, in comparison with the diseased control group, that *B. aurea—*at a dose range of 100–400 mg/kg—showed a highly significant decrease (*p* < 0.001) in blood glucose concentration. These results indicated that *B. aurea* dose-dependently produces an increase in the degree of glucose metabolism and insulin sensitivity in peripheral areas similar to that of standard hypoglycemic regimens (36). *Brugmansia aurea* also produced comparable insulin sensitivity at 200 and 400 mg/kg dose levels, suggesting its potential to increase insulin-regulated tyrosine kinase activity or insulin signaling pathways (37).

Dyslipidemia due to insulin resistance or decreased insulin secretion causes hindrance to HMG Co. A reductase enzyme (38) and also elevates the liver enzymes that cause hepatotoxicity (39). Elevated urea levels during diabetes cause diabetic nephropathy associated with impaired insulin level, protein catabolism, and oxidative damage, leading to nephrotic damage (40). *Brugmansia aurea* showed improvement in lipid profile, liver enzymes, and urea levels, suggesting that plant extracts could improve biochemical profiles during diabetes either by reducing free acid peroxidation, oxidation, or inflammation (41).

The SOD enzyme is responsible for the conversion of superoxide anion (${\text{O}}_{2}^{-}$) into H_2_O_2_ through reduction. H_2_O_2_ is then converted to water (H_2_O) and oxygen (O_2_) in mammalian cells by CAT and GSH through reduction (31, 42). Reduction in SOD causes inhibition of lipid peroxidation pathway, while a reduction in CAT and GSH leads to accumulation of H_2_O_2_, leading to oxidative damage. *Brugmansia aurea* at a higher dose (400 mg/kg) showed a significant reduction in oxidative stress by elevating the levels of endogenous antioxidants and their level of elevation is almost similar to the standard drugs. During the disease, the MDA level elevates, indicating oxidative stress exacerbates, indicating lipid peroxidation (43). *Brugmansia aurea* at 400 mg/kg dose showed similar results as standard hypoglycemic agents in terms of reducing MDA.

Improvement in HbA1c of all standard drugs and *B. aurea* doses decreased the uptake of glucose by red blood cells, thereby enhancing glucose absorption by the cells through improved insulin action (36). Increased production of insulin to cope with overt glucose intolerance causes higher fasting hyperinsulinemia in diabetic individuals, leading to insulin resistance (3, 44–47). Progressive damage to β-cells during the late phase of the disease resulted in insulin resistance and insulin deficiency (48). Higher fasting insulinemia showed higher insulin resistance, and lower levels assured lower resistance (49). *Brugmansia aurea* at 400 mg/kg produced approximately similar results in managing insulin resistance during diabetes as other standard drugs, thus improving insulin action at the cellular level.

This antidiabetic potential of *B. aurea* could be attributed to the presence of various bioactive compounds that have antioxidant and antidiabetic activity, as shown in the GC-MS and LCMS analyses of the plant extract. The most abundant bioactive compounds shown by GC/MS are 9,12-Octadecadienoic acid methyl ester and 9,12,15-Octadecatrienoic acid and methyl ester, which could be contributing to its antioxidant and antidiabetic activities. 9,12-octadecadienoic acid, also called linoleic acid, has antioxidant (50), hepatoprotective, and antiandrogenic (51). 9,12,15-octadecatrienoic acid (α linolenic acid (ALA) has anti-hypocholesterolemic, antiandrogenic, and antidiabetic activities (52–56) and inhibits reactive oxygen species (ROS) generation (52). Hexadecanoic acid, 2-hydroxy-1-(hydroxymethyl) ethyl ester, have antioxidant and anti-inflammatory activities (57).

## Conclusion

Diabetes mellitus is characterized by a combination of several abnormalities. The predominant ones are impaired glucose tolerance due to imbalanced insulin secretion and insulin resistance caused by a lack of peripheral glucose uptake. Efforts should be made to preserve β-cell function and improve insulin resistance for effective management of diabetes. This study confirmed that leaves of *B. aurea* at a high dose (400 mg/kg) concentration have beneficial effects on diabetes and its related complications, i.e., hepatic and renal function abnormalities, lipid metabolism, antioxidant enzyme levels, and especially insulin sensitivity and resistance equivalent to biguanides. The present study may provide the basis for future research on the plant to affirm the corresponding results in the management of diabetes and insulin resistance.

## Data availability statement

The raw data supporting the conclusions of this article will be made available by the authors, without undue reservation.

## Ethics statement

All experiments and procedures were approved by the REC/RIPS-LHR (Research Ethical Committee of Riphah International University Lahore) under the authorization number of Ref. No. REC/RIPS-LHR/2022053. Written informed consent was obtained from the owners for the participation of their animals in this study.

## Author contributions

NF, HA, and IS conducted the experiments. FA and BA designed the project and drafted the manuscript. AK helped in the critical revision of the drafted manuscript and data analysis. US helped with data analysis. All authors approved the final version of the manuscript.

## Conflict of interest

The authors declare that the research was conducted in the absence of any commercial or financial relationships that could be construed as a potential conflict of interest.

## Publisher's note

All claims expressed in this article are solely those of the authors and do not necessarily represent those of their affiliated organizations, or those of the publisher, the editors and the reviewers. Any product that may be evaluated in this article, or claim that may be made by its manufacturer, is not guaranteed or endorsed by the publisher.
